# Silver nanoparticles as a potential nematicide against *Meloidogyne graminicola*

**DOI:** 10.21307/jofnem-2020-002

**Published:** 2020-03-17

**Authors:** Richa Baronia, Puneet Kumar, S. P. Singh, R. K. Walia

**Affiliations:** 1 Indian Reference Materials Division, CSIR – National Physical Laboratory, New Delhi, 110012, India; 2 All India Coordinated Research Project on Nematodes in Agriculture, ICAR – Indian Agricultural Research Institute, New Delhi, 110012, India

**Keywords:** Management, *Meloidogyne graminicola*, Nematicide, Rice, Root-knot nematode, Silver nanoparticles.

## Abstract

Plant-parasitic nematodes cause severe damage to the various agricultural crops, leading to economic losses for farmers. Therefore, identification and development of novel and environmentally benign nematicides is critically important. In this study, a silver nanoparticle (AgNP) formulation was synthesized, characterized, and investigated as a potential nematicide against rice root-knot nematode, *Meloidogyne gramnicola*, on rice (*Oryza sativa*). A series of lab assays (water and sand screening) and glasshouse experiments (using soilless system, autoclaved soil, and naturally infested soil) were conducted to examine the nematicidal effects of AgNP. The results from lab assays revealed 0.1 μg/ml as the minimum concentration for 100% irreversible nematode mortality after 12 hr in the water screening test. However, results from the sand screening test indicated 100% nematicidal effect of AgNP at 2 μg/ml after 24 hr of incubation. In glasshouse assays in soilless system of rice cultivation, 1 μg/ml concentration of AgNP applied directly to the trays achieved significant suppression of root gall formation. The effective dosage to kill nematodes in field soil assays was determined to be 3 μg/ml, which is lower than the value of 150 μg/ml reported in the literature. No visible adverse effect of AgNP was observed on seed germination or plant growth in all the experiments. The results indicate that AgNP has effective nematicidal activity against *M. graminicola* in rice.

Rice (*Oryza sativa*) is a major life-sustaining crop in India and feeds more than 60% of the population. During 2014 to 2015, the area under rice crop in India was 43.86 million ha with a total rice production of 105.48 million tons (Anonymous, 2016). Rice root-knot nematode, *Meloidogyne graminicola*, is widely distributed across diverse agro-climatic conditions in India (Salalia et al., 2017) and it has emerged as the economically most threatening plant-parasitic nematode in rice nurseries causing a yield loss of USD 350 million annually (Walia et al., 2018). The current management options for *M. graminicola* focus on rice nursery bed treatments. These include summer solarization of nursery beds for 15 days using transparent polythene sheet or chemical treatment of nursery beds with carbofuran followed by main field treatment with carbofuran 40 days after transplanting (Walia and Khan, 2018). *M. graminicola*-resistant rice varieties are not currently available, and consistently effective biocontrol agents of plant-parasitic nematodes are few and offer limited choice. Carbofuran has been used widely in the recent times, but is slated to be phased out in near future. Environmentally benign chemical nematicides do not exist. Summer solarization of nursery beds is very effective but it is beset with certain limitations, such as being less effective in non-tropical and high rainfall regions. Moreover, rice is cultivated in multiple seasons in many areas and solarization is not possible in every season.

Nanotechnology is an emerging area that could provide potential solutions to problems encountered in agriculture (Iavicoli et al., 2017). Nano-formulations of currently used chemical and biological products are in vogue to enhance their efficacies. Silver ion (Ag^+^) and its compounds are highly toxic to microorganisms, exhibiting strong biocidal effects on many species of bacteria and fungi (Park et al., 2006; Kim et al., 2007; Lara et al., 2011). Recently, Ag nanoparticles (AgNP) have shown evidence of being a potentially effective nematicide (Cromwell et al., 2014; Abdellatif et al., 2016; Hassan et al., 2016; Nassar, 2016; Taha, 2016).

The first report on the bio-efficacy of AgNP on a plant-parasitic nematode was provided by Cromwell et al. (2014) against root-knot nematode on bermuda grass with mixed results, the lab assays revealed promising results but field experiments were not conclusive. Subsequent studies have explicitly revealed promising results of AgNP (Abdellatif et al., 2016; Hassan et al., 2016) and FeNP (Sharma et al., 2017) against *Meloidogyne incognita* on eggplant, tomato, and okra. The results were similar to fenamiphos and oxamyl at 0.003 ml/kg soil (Hassan et al., 2016). In the present study, a lab synthesized formulation of silver nanoparticles (AgNP) was tested against *Meloidogyne graminicola* in lab assays and glasshouse experiments. Besides, its efficacy was also compared with a commercial AgNP product (Silvox 500^®^).

The objectives of this project were to (i) characterize the physical properties of a citrate reduction synthesized AgNP formulation compared to a commercially available AgNP (Silvox 500^®^), (ii) determine the nematicidal concentration of lab synthesized AgNP against *M. graminicola*, and (iii) compare the nematicidal efficacy of AgNP vs Silvox 500^®^ in naturally infested soil against *M. graminicola*.

## Materials and methods

### Synthesis of AgNP

AgNP were prepared by chemical route using a citrate reduction method (Mulfinger et al., 2007). Briefly, 50 ml aqueous solution of 1 mM AgNO_3_ was heated to boiling temperature. After that, 5 ml of 10 mM trisodium citrate aqueous solution was added dropwise under vigorous stirring until the color of the solution changed to pale yellow. The stirring was continued for another 15 min, and the sample was allowed to cool to room temperature.

### Physical characterizations

UV–Visible (UV–Vis) spectroscopy was used to optically analyze the AgNP and commercially available Silvox 500^®^ formulation using UV–Vis-Near Infra-red (NIR) spectrophotometer (CARY 5000 series), Agilent technologies. The absorbance was recorded in the range of 200 to 600 nm. The X-ray diffraction (XRD) characterization was carried out using Rigaku Miniflex 600 diffractometer operated at 30 KV with CuKα radiation (*λ*=0.15418 nm). The diffraction data were recorded for 2θ angle ranging from 20 to 90° with a scan rate of 2° per min to ensure the crystalline structure of AgNP and also the particle size. The size of the AgNP was calculated using Scherrer’s formula, $D=\frac{0.9\lambda }{\beta \cos\theta }$, where *D* is the particle diameter, *λ* is the wavelength of X-rays used, *β* is the full width at half maximum (FWHM), and *θ* is the Braggs diffraction angle. The morphology and monodispersity of the AgNP were studied using transmission electron microscope (TEM). The micrographs were recorded using Tecnai G2 F30 S-Twin (FEI, Super twin lens with Cs=1.2 mm) instrument operated at accelerating voltage at 300 KV, having a point resolution of 0.2 nm and lattice resolution of 0.14 nm. The TEM samples were prepared by ultrasonic dispersion of AgNP, followed by drop-casting a small amount on a 300-mesh carbon-coated copper grid. The surface morphology of AgNP treated and untreated roots were studied using a scanning electron microscope (SEM, Model No. VP-EVO, MA-10, Carl-Zeiss, UK) equipped with energy dispersive spectrometer (EDS). The samples of both untreated and AgNP-treated roots were dried completely before subjecting to microscopic surface analysis. The AgNP estimation in parts per million (ppm or μg/ml) was determined by atomic mass absorption spectroscopy (Model: AAS, Vario 6, Analytik Jena, Germany).

### Direct exposure of AgNP to *M. graminicola* in water

A pure culture of rice root-knot nematode, *M. graminicola*, maintained on a soilless system (Kumar et al., 2017) in a glasshouse at National Phytotron Facility of ICAR-IARI, New Delhi was used in this study. Second-stage juveniles (J2) of *M. graminicola* were obtained by manually removing the root galls from infected rice plants and using modified Baermann assembly to extract live J2. In a series of lab assays, various aqueous concentrations (0.01~50 μg/ml) of lab synthesized AgNP were tested. Four assays were done starting with high concentrations (5-50 μg/ml), followed by 1 to 5 μg/ml, 0.1 to 1.0 μg/ml, and 0.01 to 0.1 μg/ml. Equivalent concentrations of trisodium citrate were also tested to ensure any nematicidal effect of the reducing agent. Aqueous suspension of nematodes was standardized to about 100 J2 per ml water. All lab assays were conducted in 32 well-pre-sterilized tissue culture plates. In all, 1 ml of nematode suspension was pipetted in each well, followed by 1 ml of double strength AgNP product. Observations were recorded using a stereozoom binocular microscope (Leica) at 40× at periodic intervals (1, 3, 6, and 12 hr), on nematode immobilization and mortality. Nematodes showing zig-zag shape but no movement were categorized as ‘Immobile’ (still alive), while the straight nematodes without any movement were recorded as ‘dead’. Three replicates were maintained for each concentration. In the control, an equal amount of water was added instead of AgNP. All plates were kept at room temperature (26 ± 2°C). After 12 hr of treatments, the nematodes were filtered with a sieve (pore size 25 μm), re-suspended in tap water, and observed after 1 hr for revival of activity, if any.

### Direct exposure of AgNP to *M. graminicola* in sand

A laboratory trial was conducted using oven sterilized (100°C) sand contained in 5-cm diameter glass petri dishes. The final water saturation level was 6 ml per 25 g sand. About 500 J2 of *M. graminicola* were added to sand in an aliquot of 3 ml of water, followed by 3 ml of double strength lab synthesized AgNP solution. In the control plates, 3 ml more water was added. All the plates were kept in a biological oxygen demand (BOD) incubator at 26 ± 2°C. Each treatment was replicated three times. Three plates were removed after 24, 48, and 72 hr of treatment, respectively. The whole content of petri plate was washed into a beaker using tap water. The contents were swirled for 1 min, sand allowed to settle for 10 sec, and the supernatant was collected in another beaker. The supernatant was used to observe the J2 under stereozoom microscope. Observations were recorded on nematode mortality.

### AgNP effects on rice grown in a soilless system

This and subsequent experiments were conducted in a glasshouse (30±2°C) at National Phytotron Facility of ICAR-IARI, New Delhi. Rice seedlings were raised in a soilless system in 10×5 cm plastic trays (Kumar et al., 2017). Double strength solutions of the lab synthesized AgNP product was poured into the trays at 10 ml per tray and 1000 J2 of *M. graminicola* in equal amount of water were also released, so as to make the final concentration of 1, 2, and 3 μg/ml of AgNP. In the control, 20 ml water was added. Ten replicates were maintained for each treatment and were completely randomized. The seedling mat from each tray was removed 25 days after nematode inoculation and the roots examined for the number of galls in the entire mat.

### AgNP effects on rice grown in autoclaved soil

Clay loam soil collected from a rice field of Genetics Division at ICAR-IARI farm was sterilized in an autoclave at 1.5 kg per sq. cm. (121.6°C) for 2 hr, cooled and filled in 10-cm plastic pots. Lab synthesized AgNP was applied as seed soaking or soil drench at 1, 2, and 3 μg/ml. For seed soaking, rice seeds were kept overnight in respective concentrations of AgNP solutions or water (control). Nematode inoculum (1000 J2 per pot) was released uniformly over the soil surface in 50 ml water suspension. For soil drenching, nematode inoculation was followed by application of equal amount of respective double strength AgNP solutions. In both cases, 100 rice seeds (cultivar Pusa Basmati 1121) were placed over the soil surface that was covered with small quantity of the same soil. The plants were maintained in the glasshouse and watered regularly. Root gall counts and fresh weight of 10 randomly selected seedlings were recorded at the time of termination (25 days from inoculation) of experiment. Four replicates were maintained for each treatment, and all the pots were arranged in a complete randomized design.

### AgNP effects on rice grown in naturally infested soil

Field soil naturally infested with *M. graminicola* was collected from the same location described above. The soil was partially air-dried, mixed thoroughly, and three samples of 200 cm^3^ each (collected randomly from the whole batch) were processed by wet sieving (Cobb, 1918; Whitehead and Hemming, 1965) to assess the initial population of *M. graminicola* J2. Plastic pots (1 kg capacity) were filled with soil, and each pot was drenched with 100 ml of the desired concentration (3, 6, 9, and 12 μg/ml) of lab synthesized AgNP or Silvox 500^®^. Untreated control pots were given water only. In total, 100 rice seeds (cultivar Pusa Basmati 1121) were placed over the soil surface and covered with small quantity of same soil. The pots were watered regularly. Observations were recorded on seed germination, seedling fresh weight, and number of galls on 10 randomly taken seedlings per pot 25 days after germination. Four replicates were maintained for each treatment, and all the pots were arranged in a complete randomized design.

### Statistical analyses

All the experiments in laboratory and glasshouse were conducted in a completely randomized design. All the experiments were conducted twice using the same methods. The data were subjected to analysis of variance (ANOVA) using statistical package of ICAR–Indian Agricultural Statistical Research Institute, New Delhi, India available online (webfmc.iasri.res.in). Least significant differences (LSD)/critical difference (CD) were calculated for each trial. Means were separated at *α* = 0.05. There were no Treatment×Trial interactions for any of the experiments; therefore, the two trials were combined for presentation.

## Results

### Characterization of AgNP and Silvox 500^®^

**Figure 1:**
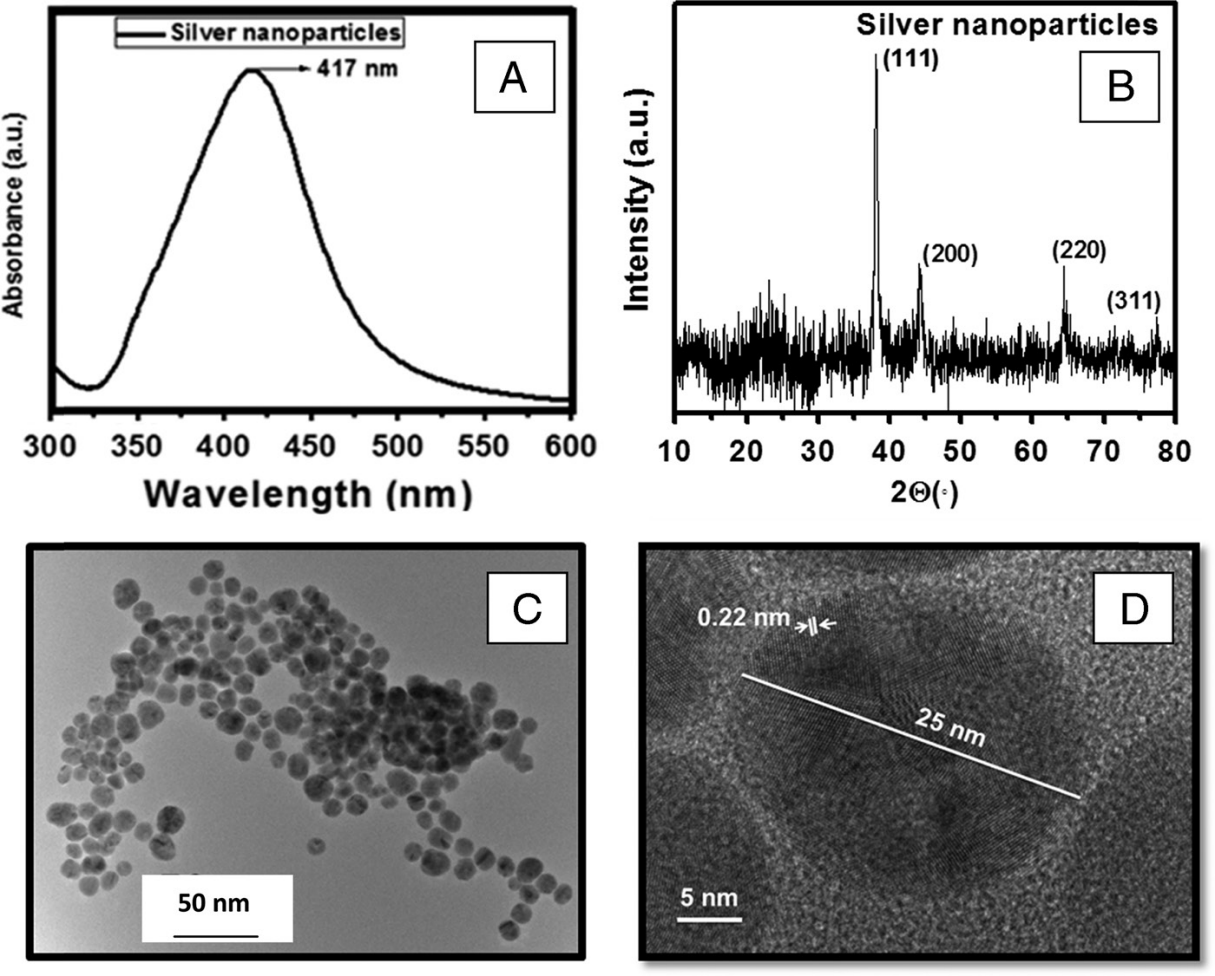
Silver nanoparticles (AgNP) characterization (A) ultra violet-visible (UV–Vis) absorption spectra exhibiting 417 nm absorbance related to surface plasmon resonance of AgNP, (B) X-ray diffractogram showing the different reflections from crystalline planes of AgNP, indicating the formation of face-centered cubic (FCC) structure of AgNP, (C) transmission electron microscopy (TEM) image showing the formation of poly-dispersed spherical AgNP with average size of 20 nm, and (D) high resolution TEM image of single AgNP of 25 nm showing characteristic inter-planar spacing of silver (Ag).

UV–Vis spectra of the AgNP revealed a well-defined surface plasmon resonance absorption peak centered at 417 nm (Fig. 1A), which is the characteristic peak related to spherical AgNP. The broadness of the absorption peak indicated the formation of poly-dispersed nanoparticles. The crystalline structure of synthesized nanoparticles was investigated using X-rays diffractometer having CuKα source. The diffraction peak at a 2*θ* value of 38.18°, 44.32°, 64.50°, and 77.05° corresponded to 111, 200, 220, and 311 planes, respectively, in regards to the face-centered cubic (FCC) structure of silver (Fig. 1B). The Bragg diffraction peak positions and their intensities are in agreement with the standard Joint Committee on Powder Diffraction Standards (file no. 04-0783) files for FCC silver. The absence of any extra diffraction plane and broadness of diffraction peaks indicated the phase purity and formation of AgNP, respectively. The particle size calculated from diffraction plane 111 was found to be ~20 nm corresponding to a d-spacing (distance between planes of atoms that give rise to diffraction peaks, each peak in a diffractogram results from a corresponding d-spacing) of 0.22 nm. The dispersion and size of the synthesized AgNP were analyzed using TEM; these were mostly spherical with an average particle size of ~20 nm (Fig. 1C,D). The high resolution TEM image revealed the d-spacing of 0.22 nm for 111 diffraction plane with a particle size of ~20 nm, corroborating the structural parameters obtained from XRD.

**Figure 2:**
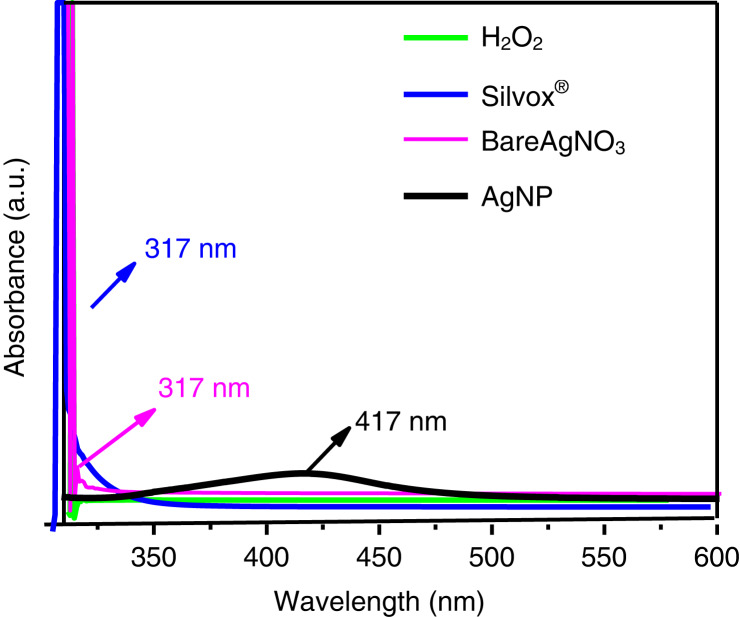
Ultra violet-visible (UV–Vis) spectra of silver nanoparticles (AgNP), AgNO_3_, H_2_O_2_, and commercial Silvox 500^®^ showing the distinctive features of AgNP which is absent in Silvox 500^®^.

Silvox 500^®^ only contained silver ions without the presence of silver nanoparticles as confirmed by lack of 417 nm peak on Silver 500^®^. Similarly, bare AgNO_3_ also did not form a peak at 417 nm in the US-Vis spectra (Fig. 2).

### Direct exposure of AgNP to *M. graminicola* in water

A benchmark of 100% nematode mortality was targeted to identify the lowest concentration of AgNP. J2 were normal up to 0.08 μg/ml concentration. Immobility of J2 was evident within 1 hr from 0.09 μg/ml concentration onwards and it increased to 100% from 3 hr onwards at 0.10 μg/ml and higher concentrations. The lowest concentration of AgNP for 100% mortality was identified as 0.1 μg/ml by 12 hr. The J2 did not recover after rinsing with water. Trisodium citrate alone had no effect on nematode immobility/mortality; therefore, the nematode mortality is attributed to AgNP exclusively.

### Direct exposure of AgNP to *M. graminicola* in sand

Unlike water screening, 0.1 μg/ml of AgNP proved ineffective even up to 72 hr (Table 1). Juvenile mortality started at 0.2 μg/ml, and it increased with increasing concentration of AgNP and time (*p* ≤ 0.05), but 100% mortality was achieved at concentrations of 2 μg/ml and above after 24 hr. The interaction of time and concentration of AgNP was significant (*p* ≤ 0.05) (Table 1).

**Table 1.** Percentage mortality of *Meloidogyne graminicola* J2 as affected by concentrations of silver nanoparticles (AgNP) in lab assays using sand as the medium.

	Percent juvenile mortality after		
AgNP conc. (μg/ml)	24 hr	48 hr	72 hr
0^a^	0	0	0
0.1	0	0	0
0.2	13.3	6.7	16.3
0.3	36.7	30	55
0.4	43.3	46.7	68.3
0.5	60	83.3	80
1.0	73.3	96.7	93.3
2.0	100	100	100
3.0	100	100	100

Notes: ^a^Control (no AgNP, water alone). Means are averages of three replications. Data were subjected to 9 × 3 (AgNP concentration × incubation time) factorial analysis of variance (ANOVA), critical difference (CD) for incubation time is 0.027, CD for AgNP concentrations is 0.468, and that for time ×  concentrations is 0.81.

### AgNP effects on rice grown in a soilless system

**Figure 3:**
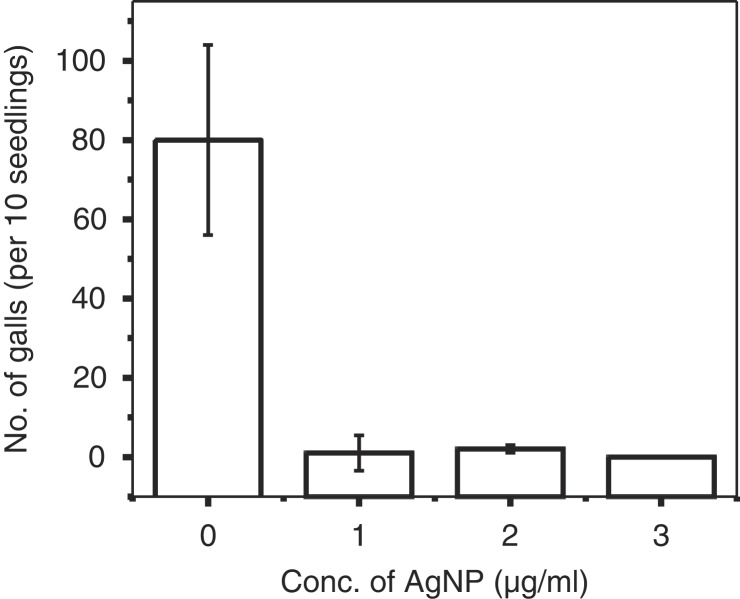
Effect of silver nanoparticles (AgNP) on root galling by *Meloidogyne graminicola* on rice seedlings in soilless system. The experiment was repeated, the Trial×Treatment interaction was not significant (*p*>0.05). Data are means of two trials. All treatments had 10 replications randomized completely.

**Figure 4:**
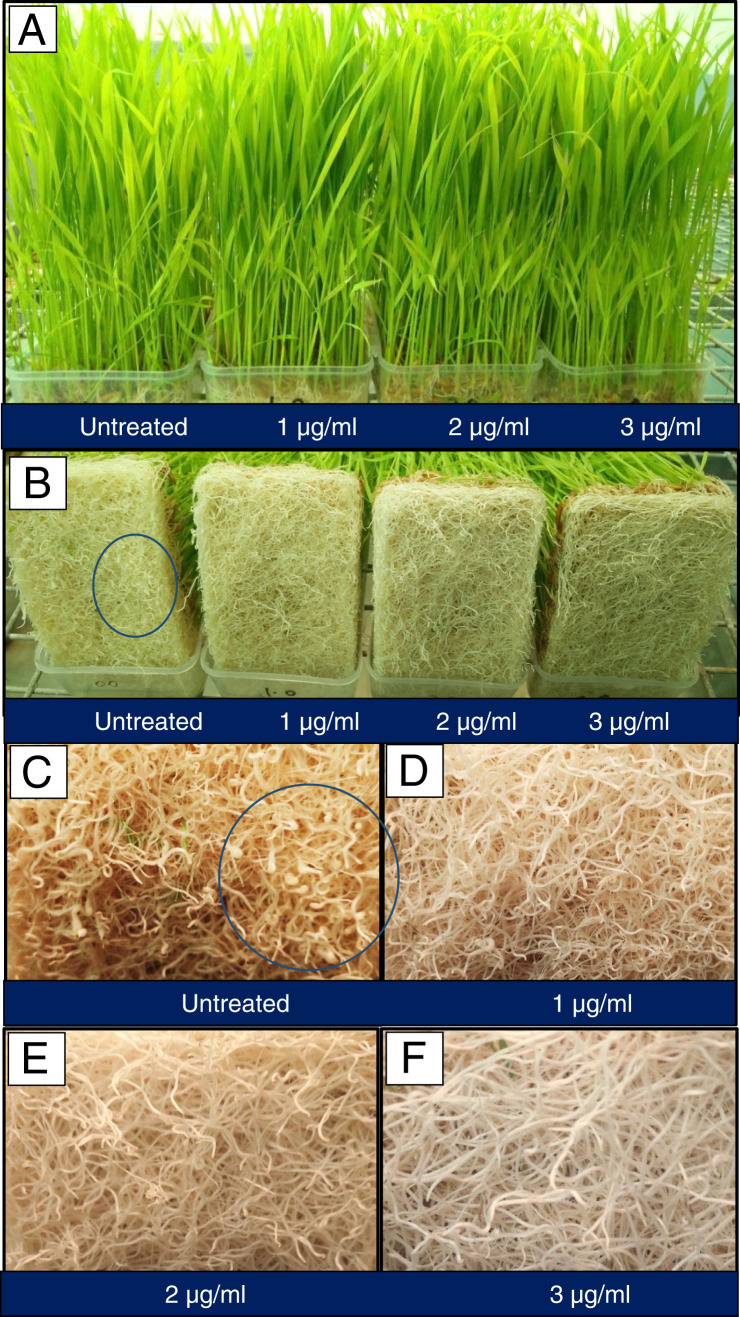
(A) Rice seedlings grown in soilless medium, (B) root mats of different treatments showing galls, and (C-F) magnified view of root mats showing galls.

**Figure 5:**
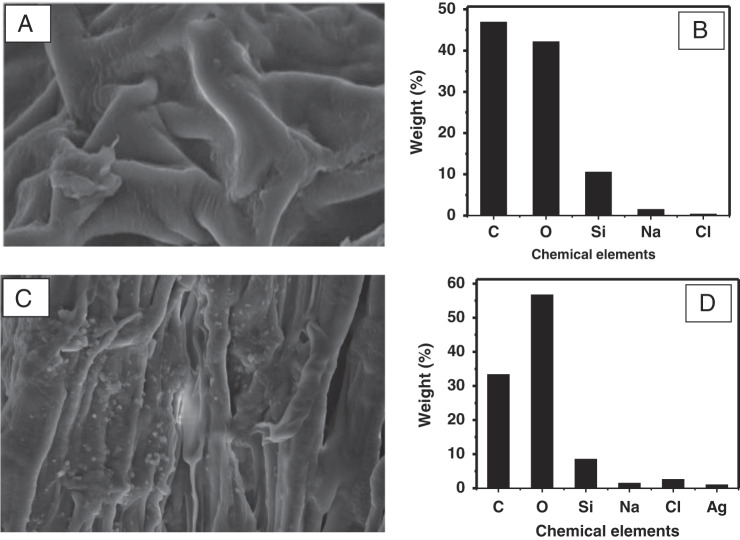
(A) Scanning electron microscope (SEM) micrographs of rice roots without silver nanoparticles (AgNP) treatment, (B) energy-dispersive X-ray spectroscopic elemental analysis of rice roots without AgNP, (C) SEM micrographs of rice roots with AgNP, and (D) energy-dispersive X-ray spectroscopic elemental analysis of rice roots with AgNP.

The AgNP treatments at 1, 2, and 3 μg/ml concentrations in soilless system were equally effective (*p* ≤ 0.05) and resulted in negligible to complete suppression of root galling compared to the control (mean 80 galls) (Figs. 3, 4). The accumulation/adherence of AgNP on root surface was observed through SEM images (Fig. 5). SEM micrographs of roots of the control samples (without AgNP treatment) did not exhibit any granular material on root surface (Fig. 5A) and EDS spectra of the control roots revealed the presence of C, O, Si, Na, and Cl, but not Ag (Fig. 5B). The presence of AgNP as granular material was visible on the treated roots (Fig. 5C). EDS spectra along with the SEM micrographs revealed the presence of Ag on the root surface suggesting the adsorption of AgNP by the roots (Fig. 5D).

### AgNP effects on rice grown in autoclaved soil

No adverse effect was observed with seed soaking in AgNP solutions for 12 hr or soil drenching with AgNP on seed germination and plant growth (Table 2). Root galling was not suppressed with AgNP at 1 μg/ml when applied to the seed or as a soil drench. AgNP at 2 and 3 μg/ml, whether applied as seed soak or soil drench were equally effective in root gall suppression compared to untreated check (*p* ≤ 0.05). Best results were obtained by applying AgNP at 3 μg/ml as soil drench (Table 2).

**Table 2.** Effect of silver nanoparticles (AgNP) as seed soak or soil drench on rice seedling growth and root galling by *Meloidogyne graminicola* in glasshouse trial using autoclaved soil.

AgNP conc.	Fresh weight of 10 seedlings (g)	No. of galls/seedling
*Seed soaking for 12 h*		
1 μg/ml	1.411a	4.96ab
2 μg/ml	1.649a	3.33b
3 μg/ml	1.291a	2.33c
*Soil drenching at 100 ml/pot*		
1 μg/ml	1.334a	6.03a
2 μg/ml	1.402a	3.70bc
3 μg/ml	1.457a	2.23c
*Untreated control*	1.277a	7.20a

Notes: The experiment was repeated, the Trial ×  Treatment interaction was not significant (p > 0.05). Data are means of two trials. All treatments had four replications randomized completely. Values with same letters in a column denote non-significant differences according to test of significance (p ≤ 0.05) in a completely randomized design.

### AgNP effects on rice grown in nematode-infested soil

AgNP, at all the doses reduced root galling significantly compared to untreated control. Although, all the doses of AgNP were statistically similar, a gradual reduction in galling was recorded with increasing dose. Silvox 500^®^ was not effective in terms of gall reduction and was similar to untreated control. Fresh weight of seedlings was not significantly different among all treatments (Table 3).

**Table 3.** Effect of silver nanoparticles (AgNP) as soil drench on rice seedling growth and root galling by *Meloidogyne graminicola* in nematode-infested field soil.

AgNP conc.	Fresh weight of 10 seedlings (g)	No. of galls/seedling
3 μg/ml	1.637a	4.9bc
6 μg/ml	1.365a	4.3bc
9 μg/ml	1.679a	3.9bc
12 μg/ml	1.318a	2.8c
Silvox^®^ solution	1.567a	5.4ab
Untreated control	1.600a	7.5a

Notes: Drenching at 100 ml per pot. Initial nematode population = 1 J2 per g soil. The experiment was repeated, the Trial × Treatment interaction was not significant (p > 0.05). Data are means of two trials. All treatments had four replications randomized completely. Values with same letters in a column denote non-significant differences according to test of significance (p ≤ 0.05) in a completely randomized design.

## Discussion

In this study, laboratory assays involving direct exposure in water unequivocally established strong nematicidal properties of synthesized AgNP at a low concentration of 0.1 μg/ml killing 100% J2 of *M. graminicola* irreversibly within 12 hr. This is lower compared to 150 and 200 μg/ml, with death rates of 80 and 91%, respectively, following 48 hr of exposure against *Panagrellus redivivus* (Mahmoud et al., 2016). Taha (2016) reported 1500 μg/ml as the most effective concentration causing 89 to 96.5% mortality of *M. incognita* J2 in lab assays after 1 to 3 days of exposure. The variability in the lethal concentrations in these studies may be attributed to procedural differences in preparation of AgNP and test nematodes.

The initial studies on the toxicity of AgNP involved free-living species *Caenorhabditis elegans* (Roh et al., 2009; Meyer et al., 2010) and *Panagrellus redivivus* (Mahmoud et al., 2016). Oxidative stress-related PMK-1 P38 MAPK activation was reported as a mechanism for toxicity of AgNP to *Caenorhabditis elegans* (Lim et al., 2012). Hassan et al. (2016) observed degradation in cell wall of *M. incognita* J2 under lab conditions. However, the exact mechanism of nematicidal effect of AgNP is unclear.

In sand screening tests, the lethal dose was 20 times higher (2 μg/ml) compared to water screening. This could be due to the adsorption of AgNP by soil particles which is expected to hinder the contact of AgNP with nematodes (Klitzke et al., 2015). This is further established by the *in planta* experiment conducted in a soilless system, where again the AgNP produced almost no galling at 1 μg/ml and above (lower concentrations were not evaluated). In glasshouse experiments using sterilized or naturally infested soil, the effective dose for gall reduction was 2 μg/ml and above. Nevertheless, even at 2 μg/ml and above in experiments using field soil, the concentration is still minimal compared to 200 μg/ml reported earlier (Mahmoud et al., 2016).

Nano-products of chemical and biological pesticides have proved to be more efficacious in combating pests and diseases leading to reduced dosages and are therefore, more economical. Some commercial products like Silvox 500^®^ are being marketed as nano-formulation with a blend of hydrogen peroxide. However, the current studies examining the particle size using UV–Vis NIR spectrophotometer did not support Silvox^®^ to be a true nano-product. The efficacy of Silvox 500^®^ (when used at equivalent silver nitrate basis in the product) was negligible in terms of gall reduction in comparative study with AgNP synthesized in this study.

It is, therefore, concluded that AgNP synthesized by citrate reduction and tested in this study may prove to be very promising at a dosage of 3 μg/ml, and further field studies are warranted. Though application of AgNP by seed dip and soil drench in nursery were equally effective, both have their own merits. While seed dipping can save the quantities of chemical used, soil drenching can ward off infection by soil-borne pathogens as well. This study has opened up new vistas in the application of AgNP for nematode management in crop plants. However, its safety to the environment needs further study. Ultimately, AgNP may provide an alternative to currently used chemical nematicides and resolve nematode problems on other crops.
